# Transglabellar Butterfly Incision for Anterior Cranial Vault Access: Case Report

**DOI:** 10.3390/curroncol31090387

**Published:** 2024-09-05

**Authors:** Jure Urbančič, Roman Bošnjak, Domen Vozel

**Affiliations:** 1 Department of Otorhinolaryngology and Cervicofacial Surgery, University Medical Centre Ljubljana, Zaloška 2, 1000 Ljubljana, Slovenia; 2 Faculty of Medicine, University of Ljubljana, Vrazov trg 2, 1000 Ljubljana, Slovenia; 3 Department of Neurosurgery, University Medical Centre Ljubljana, Zaloška 2, 1000 Ljubljana, Slovenia

**Keywords:** skull base surgery, neurosurgical procedures, craniofacial surgery, glioma resection, sinus preservation, oligodendroglioma

## Abstract

(1) Background: The transglabellar approach, a type of transfacial technique, typically involves glabellar resection and opening the frontal sinus via a bicoronal incision, providing access to the anterior cranial vault. To prevent complications, the frontal sinus is typically obliterated. However, the success of transnasal endoscopic techniques has prompted a re-evaluation of these traditional methods. (2) Methods: This paper provides a brief literature review and discusses the removal of an elongated glioma of the left gyrus rectus (4.4 × 1.9 × 2.2 cm) in a 63-year-old male using a transglabellar subfrontal approach via a butterfly incision, with frontal sinus preservation. (3) Results: An uneventful gross-total resection of a WHO grade II oligodendroglioma was achieved. There is a paucity of literature describing a transglabellar subfrontal approach via a butterfly incision with frontal sinus preservation. (4) Conclusions: The described approach could be utilized in selected cases such as small intra-axial lesions oriented longitudinally along the inferomedial frontal lobe from the posterior wall of the frontal sinus to the anterior communicating artery complex in patients with pre-existing glabellar rhytids. Since this is merely a case presentation, we cannot conclude that this represents established clinical practice. The outcomes of this approach should be investigated in the future.

## 1. Introduction

Approaches to the anterior cranial base and intracranial space can traditionally be divided into transcranial, transfacial, and transnasal routes. Each of them is expanded or adopted by one specialty [1]. Transcranial approaches are neurosurgical, whereas transfacial and transnasal approaches also fall within the domain of otorhinolaryngology due to the knowledge of the anatomy and function of the nose, paranasal sinuses, and face. A multidisciplinary team dealing with skull base diseases must be familiar with these approaches to achieve the best outcomes. The choice of skull base approach depends on the patient (e.g., age, gender, comorbidities, and anatomical characteristics), the surgeon (experience with specific approaches), and the disease (e.g., invasiveness, the location of the lesion, and the involvement of neurovascular structures) [2].

Transfacial approaches are those performed through facial skin incisions. Historically, these approaches were more critical and frequently used in the surgical treatment of diseases of the nose and paranasal sinuses [3]. Currently, their use is mainly limited to the surgical treatment of facial skeleton fractures [4].

The transglabellar approach is a transfacial approach involving the resection of the glabellar bone and opening of the frontal sinus. This approach is traditionally performed via a bicoronal incision. Access to the subfrontal lobe of the brain and the anterior cranial base (including the sella turcica and parasellar area) can be achieved through the posterior table of the frontal sinus. Thus, this approach is also known as the transglabellar transfrontal sinus subfrontal approach. Traditionally, resection of the posterior table of the frontal sinus is followed by its cranialization or obliteration with various autologous (e.g., fat, pericranial flap, or temporal muscle fascia) and heterologous materials (surgical adhesives). The purpose of obliteration is to prevent postoperative complications [5].

Due to the success of minimally invasive approaches through natural openings (i.e., without facial incisions and visible surgical scars), especially in transnasal endoscopic approaches to intradural skull base tumors, the attitude toward transfacial and transcranial approaches has changed (Wang et al., 2019 [6]). Transfacial approaches can be avoided, and many transcranial approaches can be replaced with key-hole trephination or mini-trephination variants when feasible to minimize tissue trauma and incision, lower complication rates, and shorten the recovery (e.g., mini-personal vs. personal; supraorbital vs. lateral subfrontal approach). Due to the desire to reduce morbidity associated with the use of a bicoronal flap, to avoid ear-to-ear skin incision and frontal sinus obliteration, we present the case of a patient with a low-grade glioma of gyrus rectus, in whom tumor resection along its longitudinal axis was performed using a transglabellar subfrontal approach through a butterfly incision, preserving the frontal sinus. This article aims to present the surgical technique, tricks, and precautions of the procedure, as well as potential pros and cons for such an approach.

## 2. Case Report

### 2.1. History and Clinical Findings

**Figure 1:**
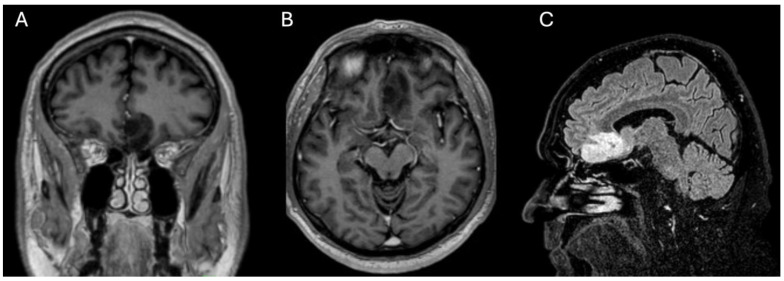
Head magnetic resonance (MRI) imaging of a patient with a low-grade glioma of left orbitofrontal cortex. (**A**)—coronal T1 contrast-enhanced MRI; (**B**)—axial T1 contrast-enhanced MRI; (**C**)—sagittal 3D brain volume imaging with enhanced water (VIEW) fluid-attenuated inversion recovery (FLAIR).

A 63-year-old right-handed man was first examined in 2015 for an intra-axial non-contrast-enhancing tumor in the left orbitofrontal lobe of the brain (a 3 × 1.5 × 1.2 cm low-grade glioma of the gyrus rectus), discovered through head imaging (MRI) due to chronic headache. Over the years of regular follow-up, the patient was refusing stereotactic needle biopsy or open navigated cytoreductive surgery. Radiological follow-up showed slow but steady tumor growth over the years, especially in the last two years. The resection of the suspected non-contrast-enhancing glioma of 4.4 × 1.9 × 2.2 cm was indicated (Figure 1). The patient had no comorbidities and was not on regular pharmacological therapy. The patient’s neurological status was normal. The patient did not report recent subjective impairments in smell and/or taste. He had no frequent infections of the nose and paranasal sinuses.

### 2.2. Surgical Procedure

**Figure 2:**
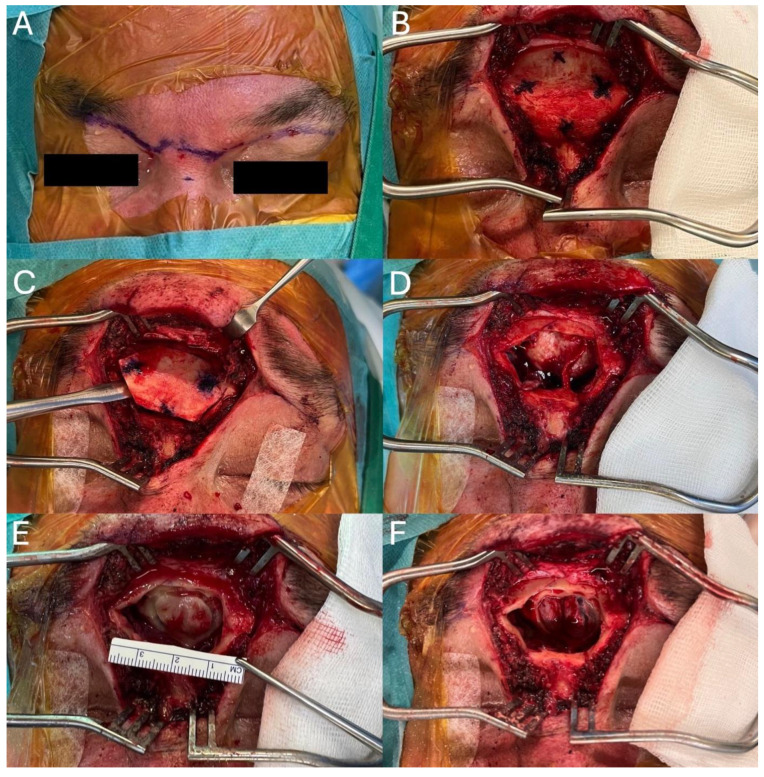
Key steps of the transglabellar subfrontal approach through a butterfly incision in a low-grade orbitofrontal lobe glioma patient. (**A**)—marked butterfly incision; (**B**)—glabella, forehead, and nasion bone marked for osteotomies after incision and dissection in subperiosteal plane; (**C**)—removal of the anterior table of the frontal sinus with a chisel after osteotomies were performed with a saw and interfrontal sinus septum blindly out-fractured; (**D**)—frontal sinus after the removal of the anterior table and retraction of sinus mucosa; (**E**)—osteotomies of the posterior table of the frontal sinus performed with a thin diamond burr; (**F**)—frontal lobe after the removal of the posterior table of the frontal sinus. The dural incision is visible over the left frontal lobe.

After appropriate preoperative preparation, the patient underwent a three-point rigid Mayfield fixation of the head in a neutral position under general anesthesia, with a neuronavigation setup (Medtronic StealthStation S8, Jacksonville, FL, USA). Intraoperatively, the patient received antibiotic prophylaxis with cefazolin intravenously. A butterfly incision was marked on the face, from the lower edge of one eyebrow (i.e., the superior part of the upper eyelid) across the nasal root to the lower edge of the other eyebrow (Figure 2A). The incision depth reached the frontal bone. Electrocauterization of both supratrochlear arteries was performed. The supraorbital neurovascular bundle was exposed only on the left side due to the extension of the incision, and it remained intact. Subperiosteal dissection was performed to expose the nasion, glabellar bone, supraorbital area, and forehead (Figure 2B). The wound was retracted using two retractors. A hexagonal osteotomy was marked on the bone and performed with a thin perpendicular oscillating saw.

The osteotomy was tangential, not perpendicular to the bone surface, to facilitate later reconstruction (Figure 2C). The interfrontal septum was fractured in a blind fashion, and the bone fragment was stored in saline. The frontal sinus was thoroughly rinsed with saline. The mucosa of the posterior table of the frontal sinus was reflected laterally with a Freer elevator and preserved for later reconstruction. The area of the frontal recess was not violated (Figure 2D). The osteotomy of the posterior table of the frontal sinus was performed with a thin diamond drill to preserve the underlying dura and harvest the single bone fragment (Figure 2E). This fragment was removed gently with a Freer elevator, and the entire mucosa was removed. The area was thoroughly rinsed with saline, and the bone fragment from the posterior table was stored in saline. This was followed by a dural incision, which was preserved and retracted laterally (Figure 2F). Microscopic tumor resection was performed under neuronavigation control using the automated continuous tumor aspiration system (NICO Myriad^®^, NICO Corp., Indianapolis, IN, USA). The tumor was well delineated from a normal brain, greyish, soft, avascular, extending back to the A2 segment of the anterior cerebral artery, and was grossly totally removed.

After ensuring hemostasis, reconstruction was performed using the heterologous material Tachosil (Corza Medical, Westwood, MA, USA) and primary dural suturing to achieve watertight closure without cerebrospinal fluid (CSF) leakage. A layer of Tachosil was placed over this. The bone defect of the posterior table of the frontal sinus was reconstructed with the preserved fragment, put in reverse, i.e., the intracranial part placed into the frontal sinus. The fragment was covered with the frontal sinus mucosa and an additional layer of Tachosil. The glabellar bone fragment, which fit tightly into the bone gap, was placed over it. Simple interrupted sutures of the subcutaneous tissue and continuous skin sutures were performed.

### 2.3. Postoperative Period and Follow-Up

The postoperative period was uneventful. Postoperative MRI showed no significant pneumocephalus, brain edema, or intracranial hemorrhage. The sutures were removed on the seventh postoperative day. The patient developed no neurological deficits, and his sense of smell remained subjectively unchanged.

The tumor was pathophysiologically characterized as a WHO grade 2 oligodendroglioma with an IDH (isocitrate dehydrogenase) mutation, 1p19q codeletion, and MGMT (O6-methylguanine-DNA methyltransferase) gene promoter methylation. The patient received a postoperative adjuvant chemotherapy regimen consisting of procarbazine, lomustine, and vincristine (PCV).

**Figure 3:**
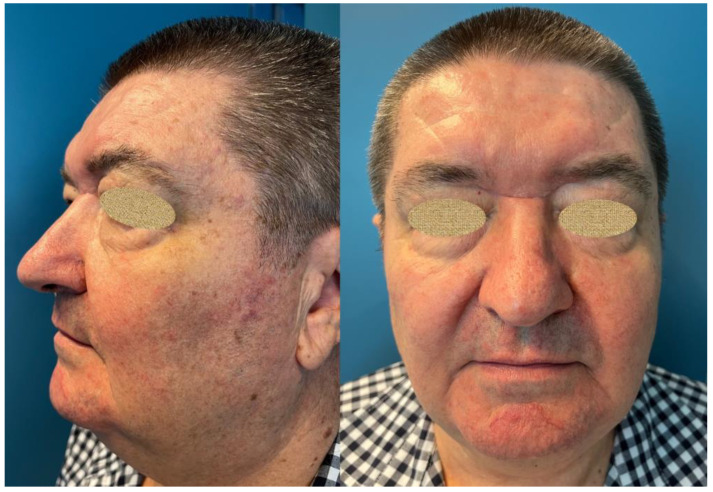
Photographs taken approximately 6 months postoperatively. Patient reported an esthetically pleasing scar along the nasal root rhytid extending bilaterally below the medial brow.

**Figure 4:**
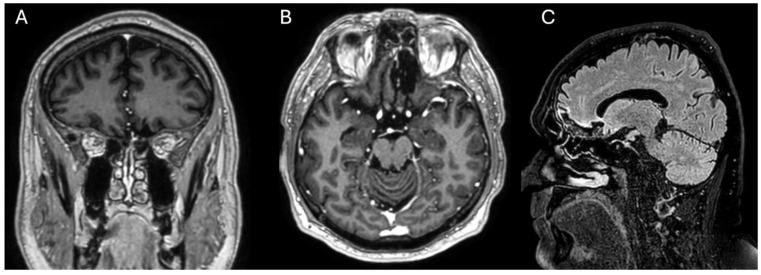
Head magnetic resonance (MRI) imaging taken approximately 6 months postoperatively. (**A**)—coronal T1 contrast-enhanced MRI; (**B**)—axial T1 contrast-enhanced MRI; (**C**)—sagittal 3D brain volume imaging with enhanced water (VIEW) fluid-attenuated inversion recovery (FLAIR). The imaging showed no residual tumor (**C**). The glioma was visually well delineated from the surrounding brain during aspiration under microscope magnification. The imaging shows good pneumatization (**C**) and mucosal lining (**B**) in the frontal sinuses.

Six months postoperatively, no complications were observed. The facial incision healed completely (Figure 3). Annual follow-up with imaging of the head and paranasal sinuses was indicated. The MRI performed approximately six months postoperatively showed no significant residual tumor (Figure 4).

## 3. Discussion

This article presents a case of a patient with a low-grade glioma of the orbitofrontal region of the brain. The tumor was resected via a butterfly incision in the glabellar area, with resection of the anterior and posterior tables of the frontal sinus, preserving the frontal sinus.

To our knowledge, such an approach to treating low-grade gliomas has yet to be described in the literature. Although we did not conduct a systematic literature review, our search found that descriptions of the subfrontal approach through the frontal sinus with obliteration predominate. Using the search key subfront*[Title] OR transglabella*[Title] OR transfront*[Title] in PubMed, we found 260 results, with only 3 describing a transglabellar approach with a butterfly incision [7,8,9]. Kleiber et al. (2014) used this approach on the frontal sinus for patients with olfactory groove meningioma, planum sphenoidale, and falx, where frontal sinus obliteration was performed [7]. Other authors performed procedures with various preservation levels, but the frontal sinus was still obliterated [10,11]. Later, this procedure was modified to treat other anterior cranial base tumors, with Al-Mousa et al. being the only one describing the preservation of the frontal sinus [8].

### 3.1. Advantages of Butterfly Incision over Bicoronal Approach

A butterfly incision for approaching the frontal sinus has many advantages over the bicoronal approach. The risk of complications is likely lower via a butterfly incision. Unlike the bicoronal incision or bicoronal flap approach, the facial nerve branches are not in the dissection field, thus preventing injury, which is the most noteworthy complication in the bicoronal approach [12]. The supraorbital neurovascular bundle is exposed only at the lateral edge of the dissection and not along its entire length, as with the bicoronal flap containing this bundle. The dissection field is smaller, reducing the risk of hematoma or seroma formation. There is no dissection along the temporal muscle in a butterfly incision, which eliminates possible postoperative temporal hollowing [12]. The risk of flap necrosis is also eliminated. A butterfly incision allows for a better aesthetic result in patients with alopecia and a receding hairline. Additionally, with a butterfly incision, there is no need to elevate the skin in areas where frontal bone osteotomy is not required. Compared with the bicoronal approach, a butterfly incision is quicker and technically less demanding [7,8,9].

### 3.2. Disadvantages of Butterfly Incision over Bicoronal Approach

In our opinion, the smaller surgical field is the drawback of a butterfly incision compared with the bicoronal approach. The supraorbital neurovascular bundles bilaterally limit the butterfly incision. The bicoronal approach fully exposes both frontal bones, which is helpful in the case of extensive frontal sinus pneumatization when obliteration is necessary. Before obliteration, all mucosa of the frontal sinus must be meticulously removed, if required, by burring [12]. In the case of a small osteotomy, mucosa removal can also be performed endoscopically with curved instruments and drills. Another disadvantage of a butterfly incision is the interruption of the supratrochlear arteries, preventing later reconstruction with a paramedian forehead flap and complicating reconstruction with a pericranial flap due to reduced blood supply. Moreover, pericranial flap elevation is technically less challenging via a bicoronal approach.

### 3.3. Frontal Sinus Preservation Misconception

Due to previous experiences, there is a misconception that in cases of posterior frontal sinus table injury with CSF fistula (traumatic or iatrogenic), obliteration or cranialization of the frontal sinus should be performed. The aim is to prevent CSF leakage with a watertight high-pressure barrier and the development of chronic frontal sinusitis by excluding communication between the frontal sinus and nasal cavities, which are not sterile. Despite obliteration, there is a risk of infection and mucocele formation years after the procedure in cases of incomplete removal of the frontal sinus mucosa [3]. Obliteration can also cause morbidity at the graft-harvesting site (e.g., abdominal fat and temporalis fascia) and prolong the surgical time. Therefore, according to recent expert opinions, obliteration is increasingly being abandoned [3]. Good results in treating posterior table frontal sinus injuries with CSF fistula can be achieved with transnasal endoscopic procedures such as Draf II and III frontotomy with skull base reconstruction, without obliteration or cranialization of the frontal sinus [13].

Cranialization is indicated in severe damage or comminution of the posterior frontal sinus table [4]. These findings support the notion that good results in treating traumatic or iatrogenic CSF fistulas can be achieved without obliteration or cranialization, as demonstrated in our case. In our case, reconstruction was performed using autologous and heterologous materials, achieving watertight closure and preserving the patency of the frontal sinus. The closure of the frontal sinus’s dura, posterior, and anterior table with the skin was multilayered, reducing the risk of postoperative CSF leakage and infection.

A finer bone cut can be alternatively performed with a piezo bone cutter or ultrasonic bone scalpel. Resorbable miniplates and screws can further improve the cosmesis if osteosynthesis is required.

### 3.4. Rationale for Transglabellar Subfrontal Approach through Butterfly Incision

The decision to utilize a transglabellar approach, in this case, was based on the tumor and the patient’s characteristics. The main argument was the mediobasal frontal location and the longitudinal growth pattern from the anterior to posterior direction. This was principally a glioma of the gyrus rectus, starting behind the frontal sinus end and extending along the midline to the anterior communicating artery (ACoA) complex. The glioma was not round, but sausage-like. The transglabellar route enabled glioma removal by navigation-controlled aspiration along its long axis without manipulating the rest of the frontal lobe.

The endonasal endoscopic approach was never thought of as an alternative due to the normal smell and, additionally, the increased risk of CSF leakage (“dead space” after tumor removal directly above the autoplasty over a large base defect).

The standard subfrontal approach would be the appropriate alternative to the transglabellar approach. This lateral subfrontal approach would necessitate repetitive elevation of the frontal lobe from the orbital roof or possibly blade retraction. The olfactory nerve, bulb, and fimbria would be set at traction. The tumor removal would be performed under dynamic retraction along its deepest aspect along the midline from the posterior wall of the frontal sinus to the ACoA complex. The superolateral aspect of the tumor would be more challenging to dissect. This approach includes the risk of retraction brain injury, rupture of the olfactory nerve, and CSF leakage from bulb–fimbria traction. Variants of lateral approaches also include the supraorbital and pterional approach with frontal extension. The pterional approach would enable adequate ACoA complex and the posterior end of the tumor exposure by proximal Sylvian fissure dissection. Anterior midline transcranial (interhemispheric) approaches with (trephination at the glabella) or without frontal sinus opening (trephination just above the frontal sinus) would necessitate a bicoronal skin incision, which we wanted to avoid, as well as frontal sinus obliteration and some brain retraction.

On the contrary, tumor removal along the long axis of the tumor by entering from the anterior (transglabellar) to the posterior direction provided a much less invasive approach, equal access to the tumor periphery from a long axis, and better visibility of the glioma–brain interface. Manipulation with the rest of the frontal lobe was avoided.

Moreover, good frontal sinus pneumatization enabled a fast approach to its posterior table without extensive drilling. Since the lesion was intra-axial and the dural layer was restored via a primary suture, there was no need for a pericranial flap, which can be readily elevated via a bicoronal approach.

The male patient had a slightly receding hairline, mild male pattern baldness, and facial rhytids along the glabella due to the prominent corrugator muscle action, which contributed to the placement of the incision along the rhytids at the glabella. Moreover, there were no noticeable complications due to the transglabellar incision, and the wound healed as a scar along the pre-existent nasal root rhytid.

### 3.5. Potential Indications of Transglabellar Subfrontal Approach through Butterfly Incision

Based on current experiences, the transglabellar subfrontal approach through a butterfly incision and frontal sinus preservation, as described in the case presented, can be indicated for small, low-grade gliomas oriented longitudinally along the inferomedial frontal lobe from the posterior wall of the frontal sinus to the ACoA complex. Removal along the long axis of the tumor avoids repetitive retraction of the frontal lobe in lateral approaches to expose the deepest medial margin of the frontal lobe. It improves the resection quality of the most challenging and accessible superior parts of the tumor. In removing the tumor along its long axis (in-line), all directions are equally accessible from the central axis toward the periphery. With the aim of neuronavigation and an innovative continuous aspiration system or simply by aspiration, the procedure technically resembles a transtubular (microscopically or endoscopically controlled [14]) microsurgery.

It is understandable that a narrower field of access results in potential difficulties when used in borderline cases. But similar to the evolution of transcranial techniques, transfacial ones may show a role for smaller keyhole approaches, regardless of their narrower range [15]. This is exactly how we understand the contemporary role of the transglabellar subfrontal approach through a butterfly incision with frontal sinus preservation.

A butterfly incision should be considered in patients with prominent facial rhytids, especially at the glabella, where the action of the procerus muscle produces horizontal rhytids. However, due to the abovementioned esthetic considerations, this approach is not suitable for children and young patients.

## 4. Conclusions

According to our experience and literature review, the transglabellar subfrontal approach through a butterfly incision with frontal sinus preservation can be suitable for treating intra-axial brain tumors of the medial orbitofrontal region in patients with pre-existing glabellar rhytids. Transcortical removal of the glioma via the transglabellar route and along its longitudinal axis prevents subfrontal elevation and retraction injury compared with the standard lateral subfrontal approach. The risk of CSF leakage is low, and the esthetic results are acceptable. Since this is merely a single case presentation, we cannot conclude that this represents established clinical practice. The outcomes of this treatment approach should be investigated in the future by including a series of cases.

## Data Availability

The data are contained within this article.
